# Piezoelectric Properties of Pb_1−x_La_x_(Zr_0.52_Ti_0.48_)_1−x/4_O_3_ Thin Films Studied by In Situ X-ray Diffraction

**DOI:** 10.3390/ma13153338

**Published:** 2020-07-27

**Authors:** Thomas W. Cornelius, Cristian Mocuta, Stéphanie Escoubas, Luiz R. M. Lima, Eudes B. Araújo, Andrei L. Kholkin, Olivier Thomas

**Affiliations:** 1 Aix Marseille Univ, Univ Toulon, CNRS, IM2NP, CEDEX 20, 13397 Marseille, France; stephanie.escoubas@im2np.fr (S.E.); olivier.thomas@im2np.fr (O.T.); 2 Synchrotron SOLEIL, L’Orme des Merisiers, Saint-Aubin-BP 48, 91192 Gif-sur-Yvette, France; cristian.mocuta@synchrotron-soleil.fr; 3 Faculty of Mechanical Engineering, University of Rio Verde (UniRV), Rio Verde 75901-970, Brazil; luizrogerio@unirv.edu.br; 4 School of Natural Sciences and Engineering, Department of Physics and Chemistry, São Paulo State University (UNESP), Ilha Solteira 15385-000, Brazil; eudes.borges@unesp.br; 5 Department of Physics & CICECO—Aveiro Institute of Materials, University of Aveiro, 3810-193 Aveiro, Portugal; kholkin@ua.pt; 6 Laboratory of Functional Low-Dimensional Structures, National University of Science and Technology MISiS, 119049 Moscow, Russia

**Keywords:** X-ray diffraction, piezoelectric properties, lanthanum-modified lead zirconate titanate (PLZT)

## Abstract

The piezoelectric properties of lanthanum-modified lead zirconate titanate Pb_1−x_La_x_(Zr_0.52_Ti_0.48_)_1−x/4_O_3_ thin films, with *x* = 0, 3 and 12 mol% La, were studied by in situ synchrotron X-ray diffraction under direct (DC) and alternating (AC) electric fields, with AC frequencies covering more than four orders of magnitude. The Bragg reflections for thin films with low lanthanum concentration exhibit a double-peak structure, indicating two contributions, whereas thin films with 12% La possess a well-defined Bragg peak with a single component. In addition, built-in electric fields are revealed for low La concentrations, while they are absent for thin films with 12% of La. For static and low frequency AC electric fields, all lanthanum-modified lead zirconate titanate thin films exhibit butterfly loops, whereas linear piezoelectric behavior is found for AC frequencies larger than 1 Hz.

## 1. Introduction

Lead zirconate titanate Pb(Zr_1−x_Ti_x_)O_3_ (PZT) has been perhaps one of the most studied ferroelectric materials, due to its excellent piezoelectric, pyroelectric, ferroelectric and dielectric properties [1]. Due to its remarkable technological importance, a wide range of Zr/Ti ratios has been investigated from both a scientific and a technological point of view. The special interest is in compositions close to the morphotropic phase boundary (MPB), occurring at around Zr/Ti ~ 52/48, because the arising monoclinic *C_m_* phase enhances the piezoelectric response [2,3]. By doping PZT with different ions, its physical properties can be radically modified, or new properties appear with potential for use in different technological devices.

Lanthanum-modified lead zirconate titanate, Pb_1−*x*_La*_x_*(Zr_1−*y*_Ti*_y_*)_1−*x/*4_O_3_ (PLZT *x*/1_−_*y*/*y*), is a material with peculiar properties, with a clear potential for technological applications [4,5]. The addition of La^3+^ to the conventional PZT system distorts the unit cell and decreases the concentration of oxygen vacancies, leading to transitions from normal ferroelectric to relaxor behavior with the increase in La content [6]. Since the discovery of PLZT in 1971, relaxor ferroelectric compositions with higher relative permittivity, spontaneous polarization and lower leakage current [7] have been widely investigated as thin films for a variety of applications, including non-volatile memory and piezoelectric devices. In contrast to ordinary ferroelectrics, whose physical properties—including ferroelectric, dielectric and piezoelectric responses—are adequately described by the Landau–Ginzburg–Devonshire theory [8], relaxor ferroelectrics escape a simple physical description, and possess a number of unique features that make them promising candidates for technological applications, such as piezoelectric transducers and light shutters. Despite several experiments and proposed theoretical models [9] to explain the origin of the relaxor phenomenon in terms of polar nanoregions—relaxor materials exhibit a diffuse phase transition, a large temperature and frequency-dependent dielectric maximum—there is no consensus between different theoretical models. Its real nature is still a subject of discussion, and the fundamental physics of the relaxors remain a fascinating puzzle [10,11]. With their complex structures and intriguing properties, relaxors represent truly a frontier of research in ferroelectrics and related materials, offering great opportunities both for fundamental breakthroughs and for technological applications. In addition to the classic relaxor PLZT 9/65/35 composition, the PLZT 12/52/48 also exhibits a relaxor behavior.

A photovoltaic effect has been reported in several ferroelectric perovskite oxides, including PZT and BaTiO_3_. It is noteworthy that the maximum photocurrent and photovoltage are also observed at different PLZT compositions [12]. Regarding compositions at around the MPB, the maximum photovoltaic properties have been reported at PLZT 3/52/48 [13]. PLZT was demonstrated to be photovoltaically active, with efficiencies in the range of 0.28%. Theoretical analyses predict that an extremely high efficiency may exist in high-quality ferroelectric ultrathin films, reaching 18.7% for 8-nm thin PLZT films, which is comparable with, or even higher than, semiconductor-based photovoltaics [13].

To study the piezoelectric properties of ferroelectric thin films, various techniques, such as piezoresponse force microscopy (PFM), laser interferometry [14] and in situ X-ray diffraction (XRD) during the application of electric fields, exist [15,16,17,18]. While PFM is a surface-sensitive tool, XRD allows for measuring the piezoelectrically-induced strain within the complete depth of a thin film, with strain resolution of 10^−4^. In addition, X-ray diffraction provides access to the piezoelectric anisotropy of the material by measuring different Bragg reflections, and thus probing grains of different orientations. This in situ technique has been used on PZT thin films to study domain switching [19], the imprint effect [20,21] and the structural evolution during imprint [22].

The present work focuses on the piezoelectric properties of PLZT *x*/52/48 thin films, with *x* = 0, 3 and 12 mol% La, studied via electrical measurements of the dielectric permittivity and the polarization as a function of applied electric fields, as well as by in situ synchrotron X-ray diffraction during the application of DC and AC electric fields with frequencies covering more than four orders of magnitude. The interrelation between PLZT compositions, characterized by their piezoelectric, photovoltaic and relaxor properties and their in situ piezoelectric response, is investigated.

## 2. Materials and Methods

Lanthanum-modified lead zirconate titanate Pb_1−*x*_La*_x_*(Zr_0.52_Ti_0.48_)_1−*x/*4_O_3_ (PLZT *x*/52/48) thin films with *x* = 0, 3 and 12 mol% La were prepared via the acetate solution route. For the preparation of the solution, stoichiometric zirconium butoxide Zr(OC_4_H_9_)_4_ (Aldrich 80%), titanium isopropoxide C_12_H_28_O_4_Ti (Fluka), lead acetate (CH_3_COO)_2_Pb.3H_2_O (Dinâmica) and lanthanum oxide La_2_O_3_ (Aldrich) precursors were added sequentially. The titanium isopropoxide was put into a beaker on a hot plate containing zirconium butoxide at room temperature under stirring for 5 min. Subsequently, 1 mL of glacial acetic acid was added to the solution at room temperature under stirring for an additional 5 min. Finally, lead acetate and lanthanum oxide were added to the solution, increasing the temperature in the sequence to 80 °C. After completing the solubilization by adding 2 mL of acetic acid and 1 mL of distilled water (~20 min), the hot plate was switched off, keeping the solution under stirring until it cooled down to room temperature. The solution was stirred throughout the process to obtain a final solution (0.4 M) that was completely transparent and stable.

For the preparation of PLZT thin films, the precursor solutions were initially deposited on Pt/TiO_2_/SiO_2_/Si(100) substrates by spin coating at 5000 rpm for 30 s, then placed on a hot plate at ~200 °C for 5 min to remove water and, in a final step, pyrolyzed in an electric furnace (Furnace EDG 1800, The Mellen Company Inc., Concord, NH, USA) at 300 °C for 10 min. The same procedure was repeated on the previously annealed film to increase the film thickness. Finally, the films were crystallized in an electric furnace (in air) at 700 °C for 30 min. The thickness of the final films was ~500 nm. The obtained films are denoted as PLZT0 (0% La), PLZT3 (3% La) and PLZT12 (12% La). Scanning electron microscopy images and energy dispersive X-ray spectra of the PLZT3 and the PLZT12 thin films can be found in Figures S1 and S2 in the Supplementary Material. For electrical measurements, circular Au top electrodes of 500 µm diameter were sputtered on the film surfaces using a homemade shadow mask. The DC electric field dependence of the dielectric permittivity ε(E) was measured using an Agilent 4284A LCR meter (Keysight Technologies, Barueri, Brazil) at 100 kHz. A modified Sawyer-Tower circuit at 10 Hz was employed to measure the polarization vs. electric field *(P-E*) hysteresis loops.

*In situ* X-ray diffraction experiments were performed at the DiffAbs beamline at SOLEIL synchrotron (St Aubin, France) [23,24]. The incident monochromatic 10 keV X-ray beam was collimated to a size of 50 µm using a pinhole placed about 20 cm upstream of the sample. This spot size ensured that the footprint of the incident X-ray beam was always smaller than the electrode diameter at a fixed incident angle of 10° used during the diffraction measurements. Thus, this configuration guarantees experiments in the central part of the electrodes where homogeneous electric fields are expected, hence avoiding any edge effects. The in situ measurements were performed with co-planar vertical diffraction geometry with the sample mounted horizontally on a *xyz* translation stage for precise (better than 1 µm) sample positioning. The diffracted X-rays were recorded using a two-dimensional hybrid pixel area detector (XPAD, ImXPAD, La Ciotat, France) with 560 × 960 pixels and a pixel size of 130 µm (details about such detectors and data conversion can be found in [25]). It was installed at a distance of 650 mm downstream from the sample, with its short dimension along the vertical direction, thus covering an angular range of about 6.5° in 2*θ*. In order to apply an electric field, one of the gold electrodes was contacted electrically using a thin gold wire with a diameter of 50 µm while another contact was taken at the Pt back electrode. The sample was then laterally scanned, generating *XY* maps with Au diffraction contrast that allow for a precise positioning of the particular (contacted) Au electrode with respect to the incident X-ray beam. Static and alternating electric fields were applied, employing an Analog Output card (model PXI 3U from ADLINK) that allows for the generation of a bipolar tension in the range of ±10 V (5 mA maximum current). For AC measurements of the piezoelectric hysteresis loops, the X-ray diffraction signal was acquired by accumulating (internally) several thousand images, each of them with very short exposure (typically 1 µs) and synchronously taken at the very same voltage during the AC cycle [24]. Once the counting statistics were sufficient, the synchronization was shifted to the next voltage point in order to describe the hysteresis loop.

## 3. Results

### 3.1. Electric Measurements

**Figure 1:**
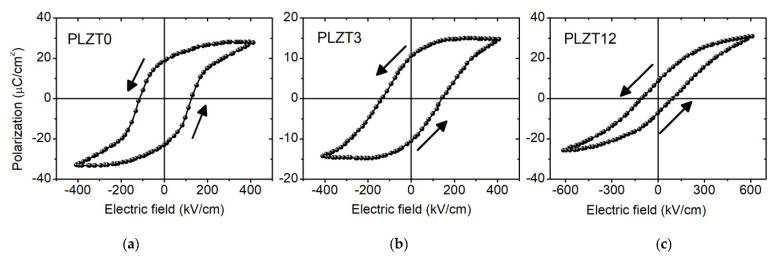
*P-E* hysteresis loops at a frequency of *f* = 10 Hz for the studied PLZT0 (**a**), PLZT3 (**b**) and PLZT12 (**c**) thin films.

Hysteresis loops of the polarization *P*, as a function of the applied electric field *E* of the studied PLZT thin films with different compositions measured at room temperature, are presented in Figure 1. The *P-E* hysteresis loops show the signature of a normal ferroelectric for PLZT0 (*x* = 0 mol% La), or PZT 52/48, while a slim hysteresis loop is observed for PLZT12, which is a typical characteristic of a relaxor material. The remanent polarizations (*P*_r_) amount to 18.7, 10.5 and 8.1 µC/cm^2^ for the PLZT0, PLZT3 and PLZT12 thin films, respectively, while the coercive fields (*E*_c_) are 125.8, 142.0 and 94.7 kV/cm for the same films. As expected, increasing lanthanum doping decreases the remanent polarization, such that the smallest *P*_r_ is observed for the relaxor composition PLZT12 (*x* = 12 mol% La). In addition, the hysteresis loops are asymmetric, and shifted towards positive electric fields, in particular for the PLZT0 and PLZT3 thin films. These asymmetries suggest a macroscopic self-polarization effect in the studied films. The asymmetries are quantified by the differences $\Delta P_{r}=P_{r}^{+}-P_{r}^{-}$ and $\Delta E_{c}=E_{c}^{+}-E_{c}^{-}$, which are given in Table 1 for the different compositions. The difference in polarization is negative, with the highest absolute value for the PLZT0 thin film, and the absolute value decreases to zero for PLZT12. The offset in the electric field is also the largest for the pure PZT thin film, and absent for the relaxor thin film. These results demonstrate that the self-polarization effect is more pronounced in PLZT0 (PZT 52/48), and tends to vanish in the relaxor composition PLZT12. The positive offset in the electric field ($\Delta E_{c}>0)$ for both PLZT0 and PLZT3 compositions indicates the presence of an internal electric field pointing towards the bottom electrode.

**Figure 2:**
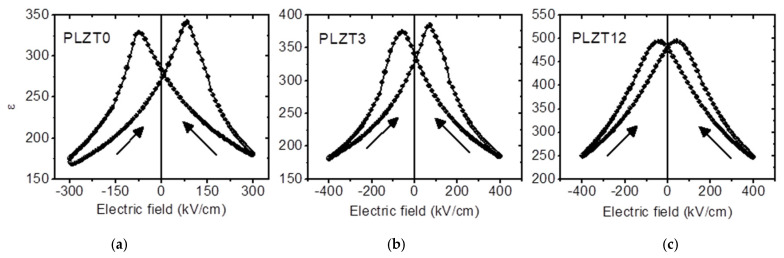
Dielectric permittivity–electric field (*ε-E*) curves for PLZT0 (**a**), PLZT3 (**b**) and PLZT12 (**c**) films with *x* = 0, 3 and 12 mol% La recorded at 100 kHz.

Besides *P-E* hysteresis loops, capacitance, as a function of electric field curves, displayed as dielectric permittivity *ε* vs. electric field *E* curves in Figure 2, is an alternative way to reveal the hysteresis in the studied PLZT thin films. The butterfly-like shapes of the *ε-E* curves also signify the polarization switching in ferroelectrics. Although the peaks in the *ε-E* curves reflect the double coercive electric field, there is some incompatibility in the values compared to the coercive fields inferred from the *P-E* hysteresis loops shown in Figure 1, because the *ε-E* curves were recorded at higher frequencies (100 kHz). An asymmetry is also observed in Figure 2 for PLZT0 and PLZT3, in contrast to the symmetric curve for the PLZT12. These data confirm the existence of macroscopic self-polarization in the studied PLZT films, which is more pronounced in the PLZT0 film and non-existent in the PLZT12 film.

### 3.2. In situ X-ray Diffraction

**Figure 3:**
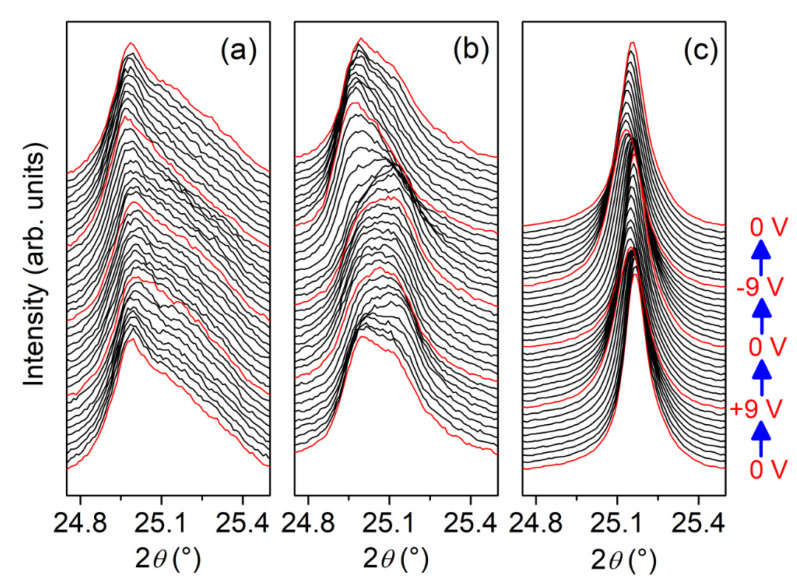
Profiles of the PLZT 110 Bragg peak for (**a**) PLZT0, (**b**) PLZT3 and (**c**) PLZT12 thin film. The profiles for different applied voltages, ranging from −9 V to +9 V, are shifted vertically for clarity. One can note the presence of double peak features for the PLZT0 and PLZT3 samples, and a simple Bragg peak for the PLZT12 thin film.

The profiles of the PLZT 110 Bragg peaks of the three different compositions of the PLZT thin films, for various voltages applied during a DC voltage sweep ranging from −9 V to +9 V, are presented in Figure 3. The profiles for the different applied voltages are vertically shifted to improve their visibility. While the Bragg reflection of the PLZT0 and PLZT3 thin films consists of at least two contributions, the PLZT12 thin film exhibits a single well-defined Bragg peak.

For high-resolution X-ray diffraction, the conventional interpretation of the double peak structure observed in Figure 3a,b would lead to the erroneous assignment of secondary phases. In the case of PZT systems, a secondary peak can be associated to the usually reported pyrochlore phase. The presence of the pyrochlore phase in PZT system is often associated to stoichiometry deviation or uncomplete perovskite growth during synthesis. However, the PLZT thin films studied in the present work were prepared under strict stoichiometric control and rigorous thin film growth protocols, leading to a very low concentration of pyrochlore phase (as demonstrated by the diffractogram shown in Figure S3 in the Supplementary Material). In addition, the main Bragg peak of the pyrochlore phase in the PZT system is observed at 2*θ* ~ 24.2° (not shown here). Hence, the observed double peak structure cannot be associated to the pyrochlore phase, but can be explained in terms of an adaptive diffraction phenomenon of the PZT monoclinic phases at the MPB, as will be discussed further below [26]. When applying an electrical potential ranging from −9 V to +9 V, the 2*θ* angle of the Bragg reflections varies.

**Figure 4:**
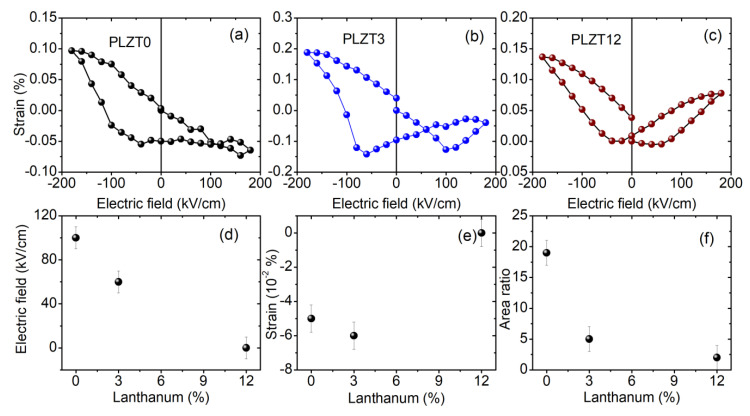
Piezoelectrically-induced strain inferred from the 2*θ*-displacement of the center of mass of the PLZT 110 Bragg reflection for PLZT thin films shown in Figure 3, as a function of the applied voltage: (**a**) PLZT0, (**b**) PLZT3 and (**c**) PLZT12. (**d**) Electric field and (**e**) strain at which the two wings of the butterfly loops presented in parts (**a**–**c**) cross each other as a function of the La concentration in the PLZT thin films. (**f**) Ratio of the areas of the left and right ‘wings’ of the butterfly loop, as a function of the La concentration.

The piezoelectrically-induced strain, inferred from the 2*θ* position of the center of mass of the Bragg reflections for the three PLZT thin films, is presented in Figure 4a–c. For all three compositions, the strain vs. electric field curve shows butterfly loops. The largest strain is obtained for the PLZT thin film with a La concentration of 3%. The cross-point where the two wings of the butterfly cross each other shifts from 0 kV/cm for a La concentration of 12% to 60 kV/cm for 3% La, and to 100 kV/cm for the pure (0% La) PZT thin film (as displayed in Figure 4d). The strain at these electric field cross-points is compressive for the pure PZT and PLZT3 thin films, amounting to −0.5% and −0.6%, respectively, while the PLZT12 thin film is almost strain-free at the cross-point of the butterfly loops (see Figure 4e). These differences go along with the asymmetry of the two wings of the butterfly loops, as also illustrated by the ratio of the areas of the two wings presented in Figure 4f, which decreases with the increasing La concentration. Asymmetric loops are generally caused by an internal bias field within the thin films, whose magnitude and distribution depend on the composition/microstructure and on the thermal/electrical history of the system.

**Figure 5:**
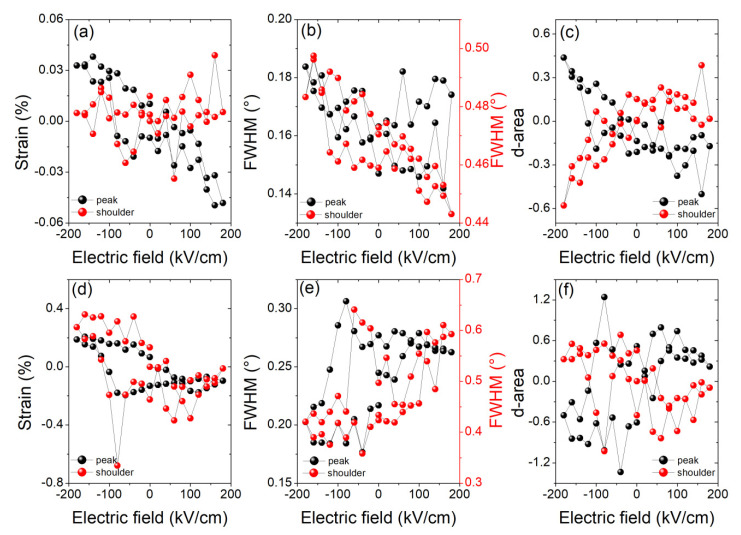
(**a**) Piezoelectrically-induced strain, (**b**) full width at half maximum (FWHM), and (**c**) variation of the area below the two pseudo-Voigt functions, used for fitting the profiles of the Bragg peaks shown in Figure 1a for the PLZT0 thin film (with respect to *E* = 0 kV/cm). (**d**) Piezoelectrically-induced strain, (**e**) full width at half maximum, and (**f**) variation of the area below the two pseudo-Voigt functions used for fitting the profiles of the Bragg peaks shown in Figure 1b for the PLZT3 thin film (with respect to *E* = 0 kV/cm).

To further analyze the two contributions of the Bragg reflection for the pure PZT thin film (PLZT0) and the PLZT3 thin film, the profiles of the PLZT 110 Bragg peaks were fitted using two pseudo-Voigt functions. The strains induced in the PLZT0 and the PLZT3 thin films by the applied electric field are presented in Figure 5a,d, respectively. While for the pure PZT thin film, only the main contribution of the Bragg reflection seems to be actually sensitive to the applied electric field, for the PLZT3 thin film both contributions show similar behaviors. However, the noise, and thus the uncertainty regarding the contribution, at larger 2*θ* angles is much larger, which might indicate that the seemingly piezoelectrically-induced strain is actually an artefact. The full width at half maximum (FWHM) for both pseudo-Voigt functions used to fit the Bragg reflections shows the same behavior as the piezoelectrically-induced strain (Figure 5b,e). Note that the FWHM is very different (factor of 2 to 3) for the two contributions. In the case of the PLZT3 thin film, the FWHM first increases when reducing the electric field to negative values, indicating an increased disorder, followed by a decreasing peak width for *E* < −100 kV/cm. This reducing peak width goes along with increasing strain, changing from compressive to tensile strain, implying the switching of domains. This particular evolution of the integrated XRD peak intensity (area) (Figure 5c,f) and its corresponding FWHM, not necessarily in phase with each other, makes the butterfly loop effect even more visible in the scattered intensity distribution (e.g., the maximum intensity of this Bragg peak, see Figure S4 of the Supplementary Material). In the case of the PLZT3 thin film, it is easily seen that both of the peaks (sharp and broad contributions) exhibit changes vs. the applied voltage.

**Figure 6:**
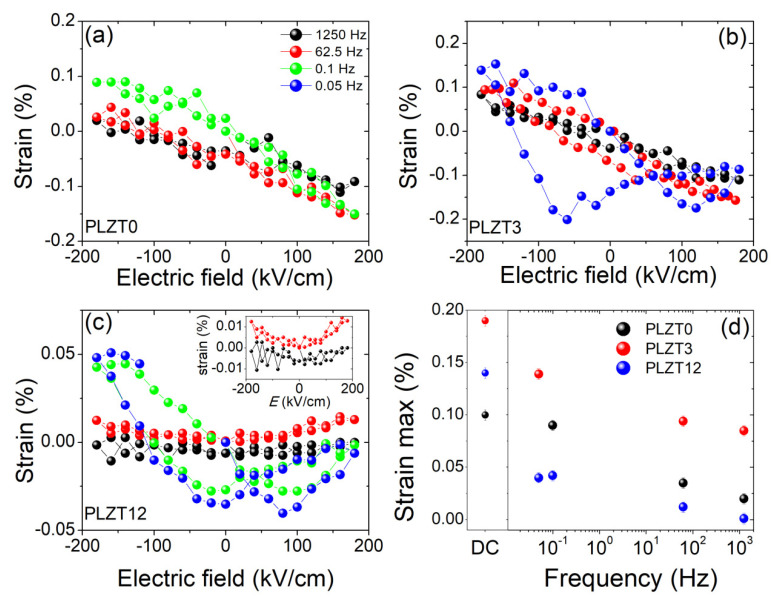
Piezoelectrically-induced strain as a function of electric field for different AC frequencies for (**a**) the PLZT0, (**b**) the PLZT3 and (**c**) the PLZT12 thin films. (**d**) Maximum piezoelectrically-induced strain at *E* = −180 kV/cm as a function of the applied AC frequency.

The piezoelectrically-induced strain in the thin films, as a function of the applied AC electric field with frequencies ranging from 0.05 Hz to 1250 Hz, is displayed in Figure 6. Due to the low intensity of the diffracted X-rays during the AC measurements, the Bragg reflections could not be reliably fitted using two pseudo-Voigt functions, and thus could not separate the two contributions. Therefore, the strain (one single component) was inferred here from the center of mass of the Bragg reflections. For the thin film with low La concentration (PLZT0 and PLZT3), a quasi-linear piezoelectric behavior is observed for AC frequencies > 1 Hz, while for very low frequencies (< 1 Hz) the shape of the hysteresis loops resembles, again, butterfly loops, and thus the piezoelectric behavior of the thin films approaches the same behavior as seen for the DC electric fields. The piezoelectrically-induced strain for the PLZT12 thin film (Figure 6c), on the other hand, does not show this linear behavior, but is closer to a typical piezoelectric hysteresis curve over the four orders of magnitude in frequency tested in the present work. As illustrated in the inset of Figure 6c, the wings of the butterfly loops for the PLZT12 thin film, at AC frequencies of 62.5 and 1250 Hz, are not apparent.

As illustrated by Figure 6d, for all three PLZT thin films studied in this work, the maximum strain measured at −180 kV/cm decreases for AC electric fields compared to DC electric fields. This decrease is particularly pronounced for the PLZT12 sample, where the strain decreases by a factor of about 3, compared to a factor of about 2 for PLZT3 and 1.2 for PLZT0. In addition, it decreases with the increasing AC frequency.

## 4. Discussion

Studies of the monoclinic phases in the PZT system, usually conducted by high-resolution X-ray diffraction, remain a fascinating topic. Three types of monoclinic phases (M_A_, M_B_ and M_C_) have been reported to exist in this system around the MPB [27], as well as in similar ferroelectric systems where the presence of a MPB depends on the composition as well as on the thermal and electric histories [28]. Due to the complex phases in the PZT system, factors like internal stress, small domain sizes and the coexistence of multiple phases easily affect the diffraction patterns, and thus strongly complicate their analysis regarding the determination of the precise phases, as well as their lattice parameters and other related effects. Although the intermediate phases may be associated with rotational polarization instabilities [29], an explanation for these phases around the MPB using the classical Landau–Ginzburg–Devonshire theory is rather difficult, since the homogeneous ferroelectric phases can be satisfactorily described only if high order terms are included in the free energy expansion [30]. In addition to these difficulties, the polarization rotation model indicates that the crystallographic anisotropy of the polarization direction disappears around the MPB as a consequence of the polarization instabilities, although the polarization rotation leads to a giant piezoelectric response in these ferroelectric systems [29]. In this scenario, an adaptive ferroelectric phase model was developed for intermediate monoclinic phases near the morphotropic phase boundaries in the ferroelectrics of complex oxides, based on the conformal miniaturization of stress-accommodating tetragonal and rhombohedral microdomains that coincide with the M_C_ and M_A_ monoclinic phases [31]. In the adaptive diffraction phenomenon, contributions to the Bragg reflections are determined by the coherent superposition of waves scattered from individual twin-related nanocrystals, and they are adaptively shifted along the twin peak splitting vectors as a consequence of changes in the twin variant volume fraction [26]. Applied to the tetragonal phase, this theory could explain the intrinsic lattice parameter relationships of the monoclinic M_C_ phase also observed in lead magnesium niobate–lead titanate solid solutions (PMN-PT). On the other hand, the (001) and (110) twin planes diffract incident waves just like the monoclinic M_A_ and M_B_ phases when applied to the rhombohedral phase of nanotwin superlattices, indicating that one or more extra diffraction peaks would appear when nanodomains coexist with coarse domains [32].

Based on the above discussion, since the presence of the pyrochlore phase is discarded in the studied PLZT thin films, the double peak structure found for low lanthanum concentrations (Figure 3a,b) suggests an adaptive diffraction phenomenon, which disappears with high lanthanum content (as shown in Figure 3c). This double peak structure is thus probably composed by the inconsistent sum of the conventional peak and the corresponding new adaptive peak originating from the coexistence of nanodomains and coarse domains. With increasing lanthanum content, the tetragonality in the PZT system decreases, thus leading to a reduction of the nanodomain and coarse domain coexistence, associated with a decrease in the spontaneous polarization in the unit cell. This reasonably explains the disappearance of the double peak structure with the increasing lanthanum content. Another possibility is that the diffraction pattern of the lanthanum-doped PZT structure behaves like an adaptive diffraction of nanotwins, where scattered waves from several nanodomains overlap coherently to form a single Bragg peak [32]. However, further studies on this system are needed to corroborate or refute this assumption.

Electrical measurements, both of *P-E* hysteresis curves and *ε-E* curves, evidenced a built-in electric field in lanthanum-modified lead zirconate titianate thin films, which is the largest for pure PZT and diminishes with increasing lanthanum content. These findings were confirmed by in situ synchrotron X-ray diffraction, revealing asymmetric butterfly loops for DC electric fields. The butterfly shape of the hysteresis loops disappears for AC electric fields with frequencies larger than 1 Hz. Similarly, differences in the coercive fields inferred from the *P-E* hysteresis loops and from *ε–E* curves, which were recorded at 10 Hz and 100 kHz, respectively, were found. This frequency dependence may originate from internal fields within the thin films that have to be overcome, and thus higher electric fields would have to be applied to eventually switch the domains. The same effect may also be the origin of the reduced piezoelectrically-induced strain for AC electric fields, compared to DC electric fields. Considering that both the butterfly shape and the absolute value of the strain are recovered at very low frequencies, i.e., for quasi-static electric fields, this indicates that the piezoelectric domains are actually hindered from switching, or that the switching process is significantly slowed down. It is well-known that quenched electric fields in ferroelectric capacitors may lead to the significant slowing down of polarization switching, due to pinning and creep effects [33,34]. A transformation of the domain structure, from micrometric 90° domains to a polar nanodomain configuration, was demonstrated for bulk lanthanum-modified tetragonal PZT with increasing La content [35]. Similarly, transmission electron microscopy studies on PLZT 10/20/80 films also revealed the absence of the normal 90° domain configuration, while it was present for Pb_0.9_(Zr_0.2_Ti_0.8_)O_3_ films [36]. This transformation of the domain structure may be the origin of the frequency-dependence reported in the present work. In addition, the interaction of the 90° domain walls with point defects may contribute to higher activation voltages. Thus, future and ongoing works focus on the behavior of PLZT thin films with regard to higher electric fields, as well as the function of time, in order to elucidate the origin of the aforementioned phenomena.

## 5. Conclusions

In conclusion, the piezoelectric properties of lanthanum-modified lead zirconate titanate thin films around the morphotropic phase boundary of pure PZT, with Zr/Ti = 52/48 and with different lanthanum concentrations ranging from 0 to 12 mol%, were investigated by in situ synchrotron X-ray diffraction during the application of DC and AC electric fields with frequencies covering more than four orders of magnitude. A double-peak Bragg reflection was found for thin films with low lanthanum concentrations, which disappears for PLZT films with 12 mol% La content. This double-peak structure is attributed to the inconsistent sum of the conventional Bragg peak and the corresponding new adaptive peak, originating from the coexistence of nanodomains and coarse domains. The piezoelectrically-induced strain describes butterfly loops for all compositions, which are symmetric for *x* = 12 mol% and asymmetric for lower La concentration. These asymmetries are due to internal electric fields in the PLZT thin films, which increase with decreasing *x* and which were also evidenced by electrical measurements. While the PLZT12 thin film shows butterfly-like loops for DC as well as AC electric fields, thin films with lower La concentrations exhibit linear piezoelectric behavior for AC frequencies larger than 1 Hz, whereas butterfly loops are apparent for low AC frequencies of about 0.1 Hz. The maximum piezoelectrically-induced strain was found to decrease for AC electric fields compared to DC electric fields, and it diminishes further with increasing AC frequency.

## Figures and Tables

**Table 1:** Summary of remanent polarization ($P_{r}^{+}$ ), electric coercive field ($E_{c}^{+}$ ) and differences in remanent polarization ($\Delta P_{r}$ ) and electric coercive field ($\Delta E_{c}$ ).

Sample	$P_{r}^{+}\;(\mu C/{\mathrm{cm}}^{2})$	$E_{c}^{+}\;(\mathrm{kV}/\mathrm{cm})$	$\Delta P_{r}=P_{r}^{+}-P_{r}^{-}\;(\mu C/{\mathrm{cm}}^{2})$	$\Delta E_{c}=E_{c}^{+}-E_{c}^{-}\;(\mathrm{kV}/\mathrm{cm})$
PLZT0	18.7	125.8	−4.2	+6.3
PLZT3	10.5	142.0	−0.1	+4.7
PLZT12	8.1	94.7	0.0	0.0

## Supplementary Materials

The following are available online at https://www.mdpi.com/1996-1944/13/15/3338/s1, Figure S1: Scanning electron microscopy images of a PLZT3 and a PLZT12 thin film, Figure S2: Energy dispersive X-ray spectrum of a PLZT3 and a PLZT12 thin film, Figure S3: Diffractograms of the three PLZT thin films studied in the present work, Figure S4: Representation of the XRD profiles measured for the different applied DC voltages for (a) the PLZT0 and (b) the PLZT3 thin film.
