# Supplementary material for: Piezoelectric Properties of Pb1−xLax(Zr0.52Ti0.48)1−x/4O3 Thin Films Studied by In Situ X-ray Diffraction
Source: Materials (Basel). 2020 Jul 27;13(15):3338. doi: 10.3390/ma13153338 (PMC7435409; doi:10.3390/ma13153338)
Supplement: Supplementary file 1 [file materials-13-03338-s001.pdf]

materials

MDPI

Supplementary Material: Piezoelectric Properties of $Pb_{1-x}La_x(Zr_{0.52}Ti_{0.48})_{1-x/4}O_3$ Thin Films Studied by *In Situ* X-ray Diffraction

Thomas W. Cornelius [1,*], Cristian Mocuta [2], Stéphanie Escoubas [1], Luiz R. M. Lima [3,4], Eudes B. Araújo [4], Andrei L. Kholkin [5,6,] and Olivier Thomas [1]

1. Aix Marseille Univ, Univ Toulon, CNRS, IM2NP, 13397 Marseille CEDEX 20, France; stephanie.escoubas@im2np.fr (S.E.); olivier.thomas@im2np.fr (O.T.)
2. Synchrotron SOLEIL, L'Orme des Merisiers, Saint-Aubin-BP 48, 91192 Gif-sur-Yvette, France; cristian.mocuta@synchrotron-soleil.fr
3. Faculty of Mechanical Engineering, University of Rio Verde (UniRV), Rio Verde 75901-970, Brazil; luizrogerio@unirv.edu.br
4. School of Natural Sciences and Engineering, Department of Physics and Chemistry, São Paulo State University (UNESP), Ilha Solteira 15385-000, Brazil; eudes.borges@unesp.br
5. Department of Physics & CICECO—Aveiro Institute of Materials, University of Aveiro, 3810-193 Aveiro, Portugal; kholkin@ua.pt
6. Laboratory of Functional Low-Dimensional Structures, National University of Science and Technology MISiS, 119049 Moscow, Russia

* Correspondence: thomas.cornelius@im2np.fr

The surface morphology and composition of the PLZT thin films was investigated by scanning electron microscopy (SEM) and energy dispersive X-ray spectroscopy (EDX). The SEM images displayed in Figure S1 show grain sizes of few tens of nanometers in size for the PLZT3 and PLZT12 thin film. The surface roughness seems to be stronger pronounced for the higher La concentration.

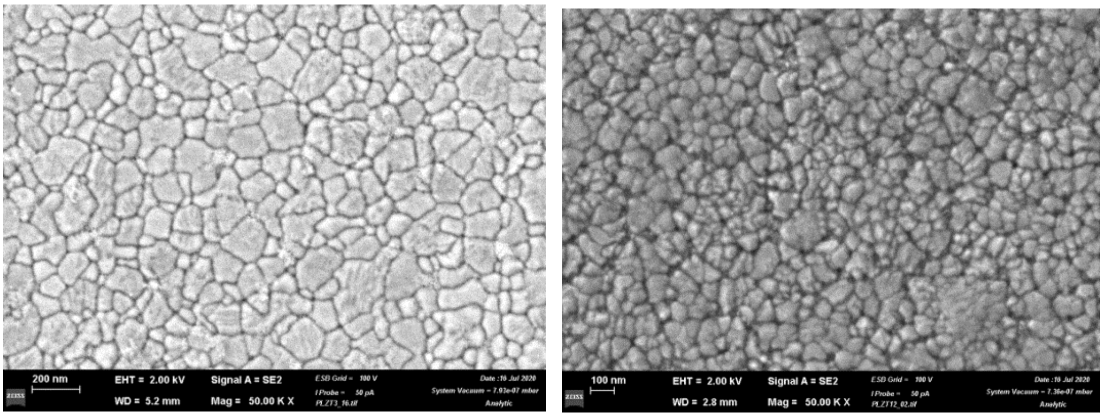

Figure S1. Scanning electron microscopy images of a PLZT3 and a PLZT12 thin film.

EDX reveals the presence of La in the PLZT3 and PLZT 12 thin film. The small amount of this element in the thin film does, however, not allow for a precise determination of the La concentrations.

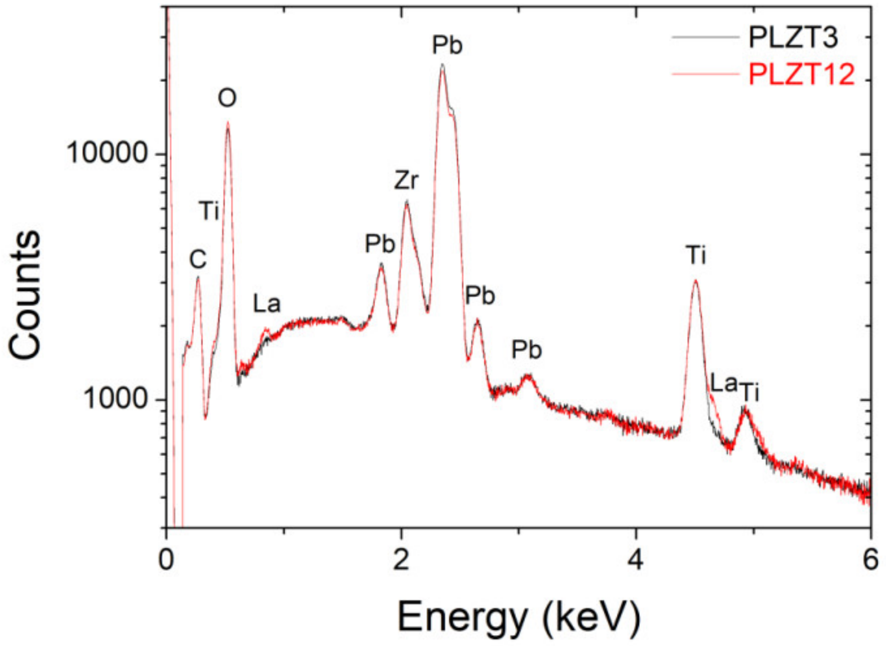

Figure S2. Energy dispersive X-ray spectrum of a PLZT3 and a PLZT12 thin film.

The structure of the PLZT thin films was examined by X-ray diffraction. The 2θ diffractogram presented in Figure S3 shows diffraction peaks from the Pt bottom electrode and the Au top electrodes. In addition, it reveals the presence of a small portion of pyrochlore phase in the PLZT thin films.

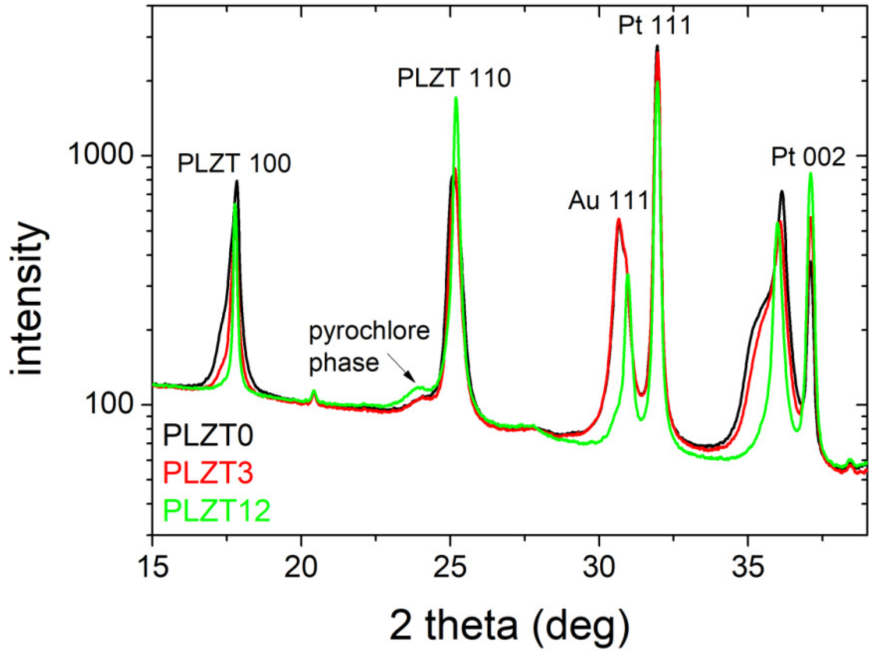

Figure S3. Diffractograms of the three PLZT thin films studied in this work.

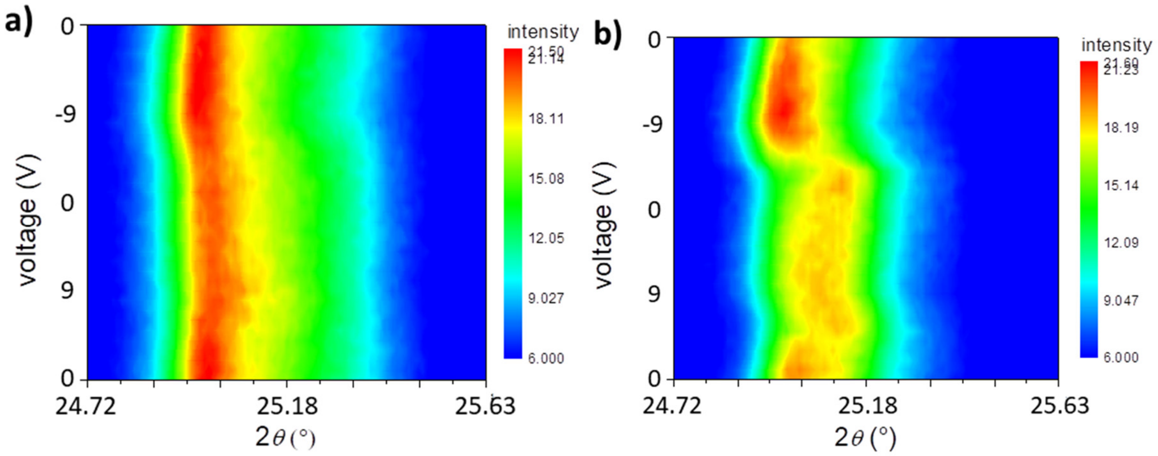

Figure S4. Representation of the XRD profiles measured for the different applied DC voltages for a) the PLZT0 and b) the PLZT3 thin film.

© 2020 by the authors. Submitted for possible open access publication under the terms and conditions of the Creative Commons Attribution (CC BY) license (http://creativecommons.org/licenses/by/4.0/).
